# Editorial: Karolinska Institutet 200-Year Anniversary Symposium on Injuries to the Spinal Cord and Peripheral Nervous System—An Update on Recent Advances in Regenerative Neuroscience

**DOI:** 10.3389/fneur.2017.00510

**Published:** 2017-09-26

**Authors:** Mattias K. Sköld, Michael G. Fehlings

**Affiliations:** ^1^Department of Neuroscience, Section of Neurosurgery, Uppsala University, Uppsala, Sweden; ^2^Department of Neuroscience, Karolinska Institutet, Stockholm, Sweden; ^3^Department of Neurosurgery, University of Toronto, Toronto, ON, Canada; ^4^Spine Program, Toronto Western Hospital, Toronto, ON, Canada

**Keywords:** spinal cord injuries, brachial plexus injury, neuronal regeneration, nerve injury, neuronal plasticity and repair

**Editorial on the Research Topic**

**Karolinska Institutet 200-Year Anniversary Symposium on Injuries to the Spinal Cord and Peripheral Nervous System—An Update on Recent Advances in Regenerative Neuroscience**

The Karolinska Institutet 200-year anniversary symposium on injuries to the spinal cord and peripheral nerves in 2010 gathered expertise in the spinal cord, spinal nerve, and peripheral nerve injury fields, covering topics from molecular prerequisites for nerve regeneration to clinical methods in nerve repair and rehabilitation (Skold et al.). The present Research Topic recognizes the remarkable advances in regenerative neuroscience that have occurred over the past years.

In this Research Topic, we are pleased to present contributions from basic laboratory studies to new clinical strategies in the spirit of highlighting advancements in regenerative neuroscience and functional repair of traumatic injuries to the spinal cord and peripheral nerves.

As the main conduits of information from the periphery to the brain and vice versa, the spinal cord and the spinal nerves are of fundamental importance. The location of spinal motor and sensory neurons within both the central and peripheral nervous systems, with profoundly different responses to nerve injury, make these neurons especially interesting for understanding fundamental aspects of nerve injury and regeneration.

In injuries to the spinal cord, the primary injury results in damage of cells, extracellular matrix, and vasculature, that in turn give rise to a secondary injury cascade with consequent ischemia, inflammation, and death of glial cells and neurons. Formation of glial scars and cystic cavities are the result of posttraumatic changes in the structural architecture of the posttraumatic spinal cord which are of importance for the capacity of regrowth of axons, the poor recovery potential and resulting neurological capacity.

The glial scar is formed in a dynamic process after injury to the spinal cord and its potentially inhibitory as well as supportive effect on nerve regrowth has been studied widely (1–4).

In the zone around the lesion, activated astrocytes, microglia, invading macrophages, and fibroblast are arranged together with secreted extracellular matrix molecules to form the glial scar. Myelin forming oligodendrocytes are commonly lost after spinal cord injuries leaving axons demyelinated (5) while surviving and new oligodendrocytes may not contribute to effective functional remyelination (6, 7). On the other hand, it is well known that oligodendrocyte progenitor cells, the so-called NG2 cells, do migrate to the spinal cord lesion and probably play a multifold function therein; secreting nerve growth inhibitory ECM molecules (chondroitin sulfate proteoglycans) and differentiating to myelin-producing oligodendrocytes and even astrocytes, although much of their function remains elusive. In the contribution to this Research Topic by Hackett and Lee, we do get a comprehensive review on NG2 cells and their role in health and disease. Hackett et al. point out that NG2 cells are, besides astrocytes, one major part of the glial scar. However, unlike astrocytes, they can differentiate into oligodendrocytes, astrocytes, and perhaps even Schwann cells and, thus, be a target in many aspects of spinal cord injury and repair.

Even though lack of nerve regeneration, or at least successful nerve regeneration, is the rule after injuries to the central nervous system (CNS), endogenous mechanisms and exceptions to this dogma of unsuccessful nerve regeneration do exist and hold promise of a wider understanding of how, when, and where nerve regeneration can occur and be supported. Anatomical and synaptic plasticity (8) as well as activation and development of neural precursor cells in to neurons and glia (9) after spinal cord injury are endogenous reparative attempts that need further exploration.

One interesting example of successful CNS nerve regeneration is the avulsion-replantation injury of spinal nerves (Carlstedt). When spinal roots, typically in high velocity traffic accidents, are torn from the spinal cord, this results in an interruption of the local transverse segmental spinal cord motor and sensory fibers. This will lead to dying back of the centrally located axon and, eventually, the motor neuron in the ventral horn of the spinal cord. However, if the avulsed spinal root is replanted to the spinal cord, survival of motor neurons and successful regeneration of axons from the motor neurons within the CNS will occur (10), which has resulted in a surgical method to restore function after this kind of longitudinal spinal cord injury (11).

In his perspectives article, Carlstedt elaborates on recent findings regarding the return of sensory function. Replantation of avulsed spinal roots leads to useful motor function if the procedure is performed before 1 month after injury (12), but sensory recovery cannot be achieved by replanting avulsed dorsal roots since the dorsal root ganglion neurons are unable to regenerate into the adult spinal cord (13).

Different strategies have been developed to overcome this problem and both use of implanted PNS conduits (14) and adjuvant therapy with a retinoic acid receptor agonist (15) has shown promising results in the restoration of sensory functions after spinal root avulsion.

In their original research paper, Bigbee et al. describe further progress in the field of sensory dysfunction after spinal root avulsion injuries in the lumbosacral plexus where replantation of ventral roots can ameliorate the otherwise resulting allodynia. In the dorsal horn after replantation of avulsed ventral roots on L6 + S1 level, they can demonstrate selective plasticity for vesicular glutamate transporter (VGLUT1) and isolectin B4 (IB4) in primary afferent projections. Given that VGLUT1 is a marker for cutaneous and muscle afferents and that IB4 is a marker for non-peptidergic primary afferents, these findings are suggestive of a restoration alternatively preservation of primary afferent phenotype expressions.

Neuronal guidance molecules are of fundamental importance in the establishment of the neuronal system during development, and a multifold of such factors and their receptors work in a complex and precisely orchestrated manner in CNS and PNS development. One such family of guidance molecules is the semaphorins (16). If and how such guidance molecules are of importance after injury and in the endogenous repair efforts in the injured CNS remains unclear and, therefore, under investigation. Previous findings in a model with regeneration of injured neurons in the spinal cord shows expression of semaphorins and their receptors in a specific pattern (17) as well as a possible interplay with growth factors related to angiogenesis (18), which is specifically interesting since vasculature and nerves share common growth factors and receptors during their establishment (19). In the contribution from Lindholm et al., the importance of the semaphorins is investigated further but now in primary sensory neurons after dorsal root injury. If such injuries are applied to the peripheral axon of the dorsal root ganglion it will be followed by vigorous regrowth, but if applied to the central part of the axon the regrowth will be much weaker. Interestingly, the expression pattern of both semaphorins and their receptors neuropilins differ distinctly between the different injuries and with specific temporal patterns, indicating an involvement in regenerative efforts of dorsal root ganglion neurites rather than inhibitory.

Anatomical variations are a common challenge, both in clinical practice and research. In their contribution to this Research Topic, Frostell et al. have made a welcomed contribution to spinal research by their effort to calculate the standard the size of the spinal cord based on 11 previous studies presenting measurements of spinal cord cross-sectional diameters. With this large and combined sample, they are able to compute population estimates of the transverse and anteroposterior diameter of the entire human spinal cord on a normalized craniocaudal axis. Information from this study will be useful in diagnosing and monitoring patients with neurodegenerative spinal disorders but also in different conditions, both in clinical and research settings, where neuronal segment relation to vertebral landmarks has to be achieved.

One important endogenous repair strategy is cerebral plasticity, i.e., the rearrangement of neuronal circuits as an answer to the new input injury (20). Thus, in surgical reconstruction of nerve injuries recovery, after such operations is a function of nerve regeneration and cerebral reorganization. In two interesting contributing original research papers and one case report, we get new insights into these mechanisms.

Dahlin et al. show in his case report an example of how the plastic capacity of the brain can be guided to improve function that has been lost in brachial plexus injuries where restoration with use of peripheral nerves, in this case phrenic nerve and intercostal nerves, have been used.

From the same group comes an original research paper (Bjorkman et al.) investigating the cerebral response to active movements in the shoulder and elbow in a group of patients with residual shoulder problems after brachial plexus birth injuries. In this study, reorganization in both contralateral and, more surprisingly, ipsilateral sensorimotor cerebral areas were demonstrated, which shows how strong the endogenous compensatory plasticity mechanisms are. Hopefully a broader understanding of this dynamic capacity of the nervous system can be used to facilitate axon regeneration in CNS injuries (21).

An example of aberrant plasticity is phantom sensation/pain after limb amputation. When amputation occurs, nerves attempt to make new connections causing reorganization within both the residual limb and the brain (22).

In their study, Collins et al. investigate if face-representing somatosensory cortical regions are able to take over the arm area in upper extremity amputees and can show that in 42% of upper extremity amputees stimulation of the face evokes phantom limb arm and hand sensations. These results demonstrate that upper limb amputation causes changes within somatosensory cortical areas, knowledge that will help understand the phantom limb pain better and holds promises for future therapeutic strategies to this debilitating condition.

We are thankful to all authors who have contributed to this Research Topic and believe that the articles offer the reader an overview of the diverse scientific approaches and latest advancements in the field of spinal cord and peripheral nerve injury and repair. It is clear from the collection of findings presented in the published papers that the progress in this field, both methodological and conceptual, will help push forward our understanding of nerve injury and repair to the benefit of our patients.

We trust that the contributions will be of interest to both basic scientists and clinical researchers and hope they will inspire further research in the fields of neurotrauma and regenerative neuroscience.

## Author Contributions

MS and MGF wrote the editorial. MS is the corresponding author.

## Conflict of Interest Statement

The authors declare that the research was conducted in the absence of any commercial or financial relationships that could be construed as a potential conflict of interest.
